# Dysregulation of complement components associated with inflammation and coagulation in virally suppressed people living with HIV

**DOI:** 10.1093/jimmun/vkaf227

**Published:** 2025-09-15

**Authors:** Natalie T Subia, Thomas K Awamura, Logan S Dean, Keona Loftis, Louie Mar Gangcuangco, Iain MacPherson, Sandra Chang, Dominic C Chow, Cecilia M Shikuma, Juwon Park

**Affiliations:** Department of Tropical Medicine, Medical Microbiology, and Pharmacology, John A. Burns School Medicine, University of Hawai’i at Mānoa, Honolulu, HI 96813, United States; Department of Tropical Medicine, Medical Microbiology, and Pharmacology, John A. Burns School Medicine, University of Hawai’i at Mānoa, Honolulu, HI 96813, United States; Department of Tropical Medicine, Medical Microbiology, and Pharmacology, John A. Burns School Medicine, University of Hawai’i at Mānoa, Honolulu, HI 96813, United States; Hawaii Center for AIDS, University of Hawai’i at Mānoa, Mānoa, HI 96813, United States; Department of Tropical Medicine, Medical Microbiology, and Pharmacology, John A. Burns School Medicine, University of Hawai’i at Mānoa, Honolulu, HI 96813, United States; Hawaii Center for AIDS, University of Hawai’i at Mānoa, Mānoa, HI 96813, United States; Department of Medicine, John A. Burns School of Medicine, University of Hawai’i at Mānoa, Honolulu, HI 96813, United States; Department of Tropical Medicine, Medical Microbiology, and Pharmacology, John A. Burns School Medicine, University of Hawai’i at Mānoa, Honolulu, HI 96813, United States; Department of Tropical Medicine, Medical Microbiology, and Pharmacology, John A. Burns School Medicine, University of Hawai’i at Mānoa, Honolulu, HI 96813, United States; Hawaii Center for AIDS, University of Hawai’i at Mānoa, Mānoa, HI 96813, United States; Department of Medicine, John A. Burns School of Medicine, University of Hawai’i at Mānoa, Honolulu, HI 96813, United States; Department of Tropical Medicine, Medical Microbiology, and Pharmacology, John A. Burns School Medicine, University of Hawai’i at Mānoa, Honolulu, HI 96813, United States; Hawaii Center for AIDS, University of Hawai’i at Mānoa, Mānoa, HI 96813, United States; Department of Medicine, John A. Burns School of Medicine, University of Hawai’i at Mānoa, Honolulu, HI 96813, United States; Department of Tropical Medicine, Medical Microbiology, and Pharmacology, John A. Burns School Medicine, University of Hawai’i at Mānoa, Honolulu, HI 96813, United States

**Keywords:** complement, platelet, neutrophil, NET, inflammation, NACM, PLWH

## Abstract

Although the interplay between the complement system, platelets, and neutrophils has been considered a major contributor to inflammation and thrombogenicity, little attention has been directed toward understanding their roles in people living with human immunodeficiency virus (PLWH). We quantified and compared expression levels of complement components (C2, C3a, C5a, C9), markers for coagulation (vWF-A2, ADAMTS13, tissue factor (TF), protein C, fibrinogen), and neutrophil activation (MPO, MMP-9) in plasma between virally suppressed PLWH (*n* = 40) and people living without HIV (PLWoH; *n* = 39). Platelet and activated platelet (CD62P^+^ cells) counts in the plasma samples were examined by flow cytometry analysis. To determine whether PLWH’s plasma promotes neutrophil extracellular traps (NETs) and whether C2 and C5a levels correlate with NET formation, an ex vivo NET assay was performed. PLWH showed significantly altered C2 and C5a levels in plasma that correlated strongly with protein C and MPO. C2 also showed a positive correlation with proinflammatory markers (SSA, SAP, IL-1β, and VEGF). Furthermore, HIV status was a significant predictor of C2 and C5a levels. CD62P expression on platelets was significantly increased in PLWH. In addition, treatment of healthy neutrophils with PLWH’s plasmapromoted NET formation, and this effect was inhibited by C5aR antibody treatment and platelet removal. These data suggest that activated platelets and soluble factors, such as higher C5a levels, contribute to NET formation in PLWH. Our findings provide evidence of complement dysregulation associated with inflammation and coagulation in PLWH. Altered soluble factors and platelet activation promote NET formation, potentially driving age-related non-AIDS comorbidities (NACMs).

## Introduction

Although effective antiretroviral therapy (ART) has drastically reduced AIDS-defining illnesses among people living with human immunodeficiency virus (PLWH), they experience disproportionately higher rates of various non-AIDS comorbidities (NACMs),^1^ such as diabetes,^2^ malignancies,^3^ and cardiovascular diseases (CVDs),^4^^,^^5^ which have emerged as significant concerns in the clinical care of PLWH.^6^ The mechanisms of NACMs are multifactorial, including traditional risk factors (smoking, hypertension, dyslipidemia, diabetes, and obesity)^7^^,^^8^ and human immunodeficiency virus (HIV)-related factors (including immunodeficiency, immune activation, and ART toxicities).^9^ Additionally, the presence and persistence of HIV reservoirs in the blood and tissues contribute to persistent immune activation and inflammation.^10^

The complement system comprises several small proteins present in plasma and on cell surfaces and plays an essential role in defense against infection.^11^ During an infection, the complement cascade typically serves as an effector arm of the innate immune response, playing a crucial role in host defense against pathogens. Activation of complement is initiated through the interaction of complement components with antigen-antibody immune complexes, mannose-binding lectin (MBL), and MBL-associated serine protease complexes, or by spontaneous hydrolysis of C3 on the surface of pathogens, leading to the formation of anaphylatoxins C3a, C4a, and C5a.^11^ Soluble C5a is a potent anaphylatoxin and acts as a chemoattractant at low nanomolar concentrations, with its cognate G-protein-coupled receptors primarily expressed on myeloid cells, such as neutrophils and macrophages. This process augments inflammation by increasing chemotaxis and the production of proinflammatory cytokines from neutrophils and macrophages.^12–14^ The complement system also contributes to shaping the adaptive immune response by promoting B cell activation and differentiation, as well as modulating T cell response.^15^^,^^16^

Although complement activation plays a critical role in HIV virion clearance, it also contributes to the spread and maintenance of the virus at different stages of HIV infection.^17^ HIV can escape complement-mediated lysis and exploit complement components to enhance infectivity and facilitate infection.^17^^,^^18^ The mechanism for increased HIV transmission to immune cells such as monocytes, macrophages, and dendritic cells, as well as non-immune cells like erythrocytes, involves the deposition of complement components C3 and C5 on the surface of HIV, allowing HIV to interact with cells expressing complement receptors.^17^

The complement system plays a central role in various inflammatory pathologies. Excessive activation or insufficient regulation of complement activation can lead to inflammatory responses and tissue injury, thereby exacerbating clinical complications.^19^^,^^20^ In HIV, a recent study by Vujkovic-Cvijin et al.^21^ showed that plasma levels of several complement components and factors were higher in PLWH than in people living without HIV (PLWoH). In addition, increased C5 levels were significantly associated with the prevalence of NACM. However, the involvement of complement dysregulation in inflammation and immune activation in PLWH is still largely unknown. This study aimed to determine and compare the levels of inflammatory cytokines and complement component factors in PLWH and well-matched PLWoH. We then determined the correlations of C2 and C5a levels with soluble proteins associated with inflammation and coagulation. Lastly, we evaluated ex vivo neutrophil extracellular trap (NET) formation induced by plasma to examine whether altered plasma soluble proteins and/or platelet activation in PLWH can promote NET formation.

## Methods

### Study subjects

This cross-sectional study analysis used archived data and banked peripheral blood mononuclear cells (PBMCs) and plasma from entry time point of the longitudinal Hawaii Aging with HIV-Cardiovascular (HAHC-CVD) study.^22^ Plasma and PBMCs were isolated as previously published^22^ and cryopreserved until further use. The HAHC-CVD study was approved by the University of Hawaii Committee on Human Subjects (CHS no. 16476 and 17857), and informed consent was obtained from all subjects.

### Measurement of complement components and soluble markers for coagulation and neutrophil activation, and cytomegalovirus (CMV) IgG

The expression levels of complement components: C2, C3a, C5a, and C9; markers of coagulation: von-Willebrand Factor-A2 (vWF-A2), ADAMTS13, tissue factor (TF), fibrinogen, and protein C; and neutrophil activation markers: matrix metalloproteinase (MMP)-9 and myeloperoxidase (MPO), were determined in the plasma samples using a Luminex bead-based multiplex immunoassay (R&D Systems Inc., no. LXSAHM). Plasma C3a levels were determined using a C3a human enzyme-linked immunosorbent assay (ELISA) kit (Thermo Fisher, no. BMS2089). The presence of immunoglobulin G (IgG) antibodies against CMV was assessed using a CMV IgG Human ELISA Kit (Abcam, no. ab10874). Absorbance was read at 450 nm using a microplate reader (SpectraMax^®^ M3, Molecular Devices, San Jose, CA). The concentration of each analyte was calculated based on the standard curve generated by the respective standards.

### *Ex vivo* NET assay

Whole blood collected from healthy volunteers was layered in a 1:1 volume ratio on 5 ml of Polymorphprep^TM^ solution and spun for 30 min at 500 × *g* without braking. The neutrophil layer was collected, and residual erythrocytes were removed by incubation with TheraPEAK^TM^ ACK Lysis buffer (Lonza Bioscience) for 5 min. Neutrophils (2.5 × 10^5^ cells/well) were seeded onto 16-mm poly-L-lysine-coated coverslips (Corning) in 12-well tissue culture plates with serum-free RPMI1640 medium and allowed to adhere at 37 °C and 5% CO2 for 10 min. To deplete platelets in the plasma, plasma samples were spun for 15 min at 1500 g to collect a platelet-poor plasma (PPP) layer. Heathy neutrophils were treated with PPP or a platelet-rich plasma (PRP), or at 5% vol/vol and incubate cells for 3 h. After fixing the cells with 4% paraformaldehyde (PFA) in HBSS for 15 min, the cells were gently washed twice with HBSS. Cells were permeabilized with 0.1% Triton X-100 in Dulbecco’s phosphate-buffered saline (DPBS) for 15 min, blocked for 1 h with blocking buffer (1.5% donkey serum, 1% bovine serum albumin [BSA] in DPBS), and incubated with primary antibodies (Histone H2B, Abcam, no. ab52484) and MPO (R&D Systems, no. AF3667) at 4 °C overnight. The cells were incubated with secondary antibodies, donkey anti-goat Alexa Fluor 594 and donkey anti-rabbit Alexa Fluor 488 (1:500 dilution, Thermo Fisher) for 1 h and counterstained with DAPI. The cells were visualized immediately using A Zeiss Axiovert 200M microscope.

### NET quantification

Neutrophils (5–10 × 10^3^ cells per well) were seeded in a 96-well plate, treated with plasma at 5% vol/vol, and incubated in a CO_2_ incubator at 37 °C for 3 h. To block the C5a-C5aR pathway, neutrophils were treated with a C5aR antibody (0.1 µg/ml) (R&D Systems, no MAB3648) for 30 min prior to incubation with PLWH plasma. After adding SYTOX Red Dead Cell Stain (Invitrogen) for 15 min at 37 °C, the endpoint fluorescence intensity (ex: 658 nm, em: 640 nm) was measured using the Varioskan Lux Multimode Microplate Reader (Thermo Fisher) and analyzed. To detect released NETs, double stranded DNA (dsDNA)was measured in the culture supernatant by a PicoGreen dsDNA Assay Kit (Thermo Fisher Scientific), and the endpoint fluorescence intensity (ex: 480 nm, em: 520 nm) was measured with the Varioskan Lux Multimode Microplate Reader (Thermo Fisher).

### Flow cytometry analysis

Platelets in plasma were stained with a titrated fluorophore-conjugated primary antibody cocktail (CD41; BioLegend, no. 303732 and CD62P; BioLegend, no. 304932) at room temperature, protected from light, for 30 min, and then washed twice with flow buffer (ice-cold DPBS supplemented with 1% BSA). Approximately 1.0 × 10^6^ events were acquired per sample on an Attune NxT Flow Cytometer (Thermo Fisher, Waltham, Massachusetts). Volumetric calculations directly determined cell counts by the Attune Nxt (Thermo Fisher, Waltham, Massachusetts) instrument. Data analysis was performed using FlowJo Version 10.8.1 (Treestar, Ashland, Oregon) software.

### Statistical analysis

An unpaired non-parametric *t* test (Mann–Whitney *U* test) was used to compare continuous variables between groups. The χ^2^ test was used to compare categorical variables between groups. Spearman’s correlation was used to compare continuous variables within a group. Continuous variables from the immunoassays that showed significant differences (*P* < 0.05) using the non-parametric *t* test (Mann–Whitney *U* test) were further analyzed using linear regression models to determine if previous categorical and continuous clinical variables can predict these values. Statistical analyses were performed in SPSS Version 29.0 and Prism 9 (GraphPad, San Diego, California), and figures were created with corresponding p-values using Prism 9.

## Results

### Study participant description

A total of 79 study subjects (PLWH: *n* = 39 and PLWoH: *n* = 40) were included in the analyses. PLWoH adults were matched for age, gender, and BMI as controls for the PLWH group. Baseline demographic characteristics of the study subjects are presented in Table 1. Overall, the PLWH and PLWoH had a median age of 51.5 and 55.3 years, respectively. The majority of both groups consisted of males (92.5% and 87.2%) and Whites (67.5% and 61.5%), respectively. Most PLWH (82.5%) had undetectable HIV RNA (HIV RNA <50 copies/mL) and a median CD4^+^ T cell count of 507.5 (424.5–706) cells/μl. Blood parameters, such as median white blood cell (WBC), absolute neutrophil, and platelet counts, were comparable between the groups. The prevalence of cytomegalovirus (CMV) coinfection was higher in PLWH than in PLWoH (*P* = 0.031). However, other comorbidities, including smoking history, hypertension, CVD, and diabetes, were not significantly different.

**Table 1.** Demographics, blood parameters, and co-morbidities in the study participants.

		PLWH (*n* = 40)	PLWoH (*n* = 39)	*P*-value
**Demographics**	Age (years)	51.5 [45.3, 57.0]	55.3 [47.5, 60.6]	0.235
	Male	37.0 (92.5)	34 (87.2)	0.433
	BMI (kg/m^2^)	26.4 [24.2, 28.8]	26.6 [22.9, 30.3]	0.837
	White	27 (67.5)	24 (61.5)	0.580
**Blood Parameters**	Undetectable HIV RNA <50 copies/ml	33 (82.5)	–	–
	CitH3 (ng/ml)	11.7 [10.8, 14.3]	–	–
	cfDNA (ug/ml)	0.783 [0.733, 0.863]	–	–
	CD4+ T-cell Count (cells/µl)	507.5 [425, 709]	–	–
	WBC count (×10^9^/l)	5.4 [4.8, 6.4]	5.1 [4.4, 5.9]	0.222
	Absolute Neutrophil Count (10^9^/l)	3.0 [2.44 3.7]	3.1 [2.2, 3.6]	0.603
	Platelet Count (×10^9^/l)	186 [164, 236]	212 [175, 242]	0.393
	Hemoglobin	14.7 [13.4, 15.3]	14.2 [13.7, 15.2]	0.624
**Co-Morbidities**	Hypertension	5 (12.5)	11 (28.2)	0.105
	High cholesterol	20 (50)	17 (43.6)	0.568
	History of myocardial infarction	2 (5)	1 (2.56)	0.571
	History of stroke	1 (2.5)	0 (0)	0.320
	Diabetes	1 (2.5)	0 (0)	0.320
	History of smoking	23 (57.5)	26 (66.7)	0.401
	Current cigarette smoking	9 (22.5)	8 (20.5)	0.830
	CMV IgG positivity^a^	26 (76.5)	20 (51.2)	0.031*

Values presented are median [quartile 1, quartile 3] and *n* (%) as appropriate.

a CMV IgG ELISA was measured in a subset of participants due to limited sample availability: PLWH (*n* = 34) and PLWoH (*n* = 39).

* Statistically significant, *P*-values using the χ^2^ test for categorical variables and the Mann–Whitney *U* test for numerical variables.

### PLWH displays altered plasma levels of complement components

We previously found that virally suppressed PLWH had higher citrullinated Histone H3 (CitH3) levels and immature low density granulocytes (LDGs) with enhanced NET formation.^23^ Recent evidence supports a role for complement activation in inducing NET formation.^24^ In turn, neutrophil activation can themselves trigger complement activation, creating a positive feedback loop that further amplifies neutrophil activation.^25^ NET formation is regulated by bidirectional interactions between neutrophils and platelets.^26^ Therefore, we investigated whether PLWH display an altered interplay of complement, platelets, and neutrophils linked to inflammation and coagulation.

**Figure 1.**
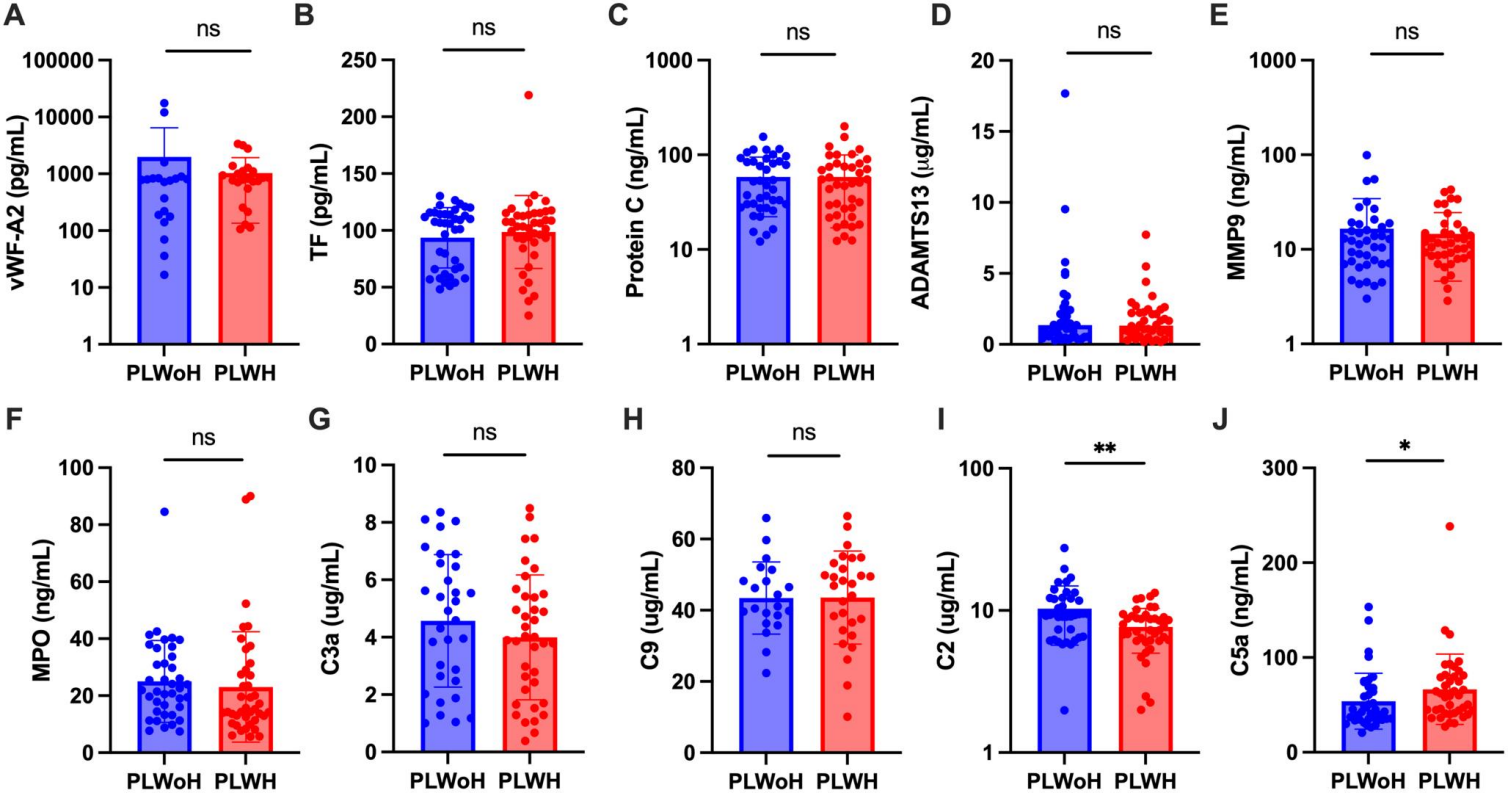
PLWH plasma displays altered concentration of complement C2 and C5a. Coagulation markers: (A) vWF-A2, (B) TF, (C) Protein C, (D) ADAMTS13, neutrophil activation markers: (E) MMP9, (F) MPO, and complement activation markers: (G) C3a and (H) C9 showed no significant difference between PLWoH (*n* = 39) and PLWH (*n* = 40) groups. Displayed a significant decrease in (I) complement C2a, but an increase in (J) complement C5a in the PLWH group, compared to the PLWoH group. Mann–Whitney *U* test for continuous variables, ns:non-significance, *P ≤ 0.05, **P ≤ 0.01.

The concentrations of coagulation markers (vWF-A2, TF, Protein C, and ADAMTS13) were not statistically different between PLWoH and PLWH groups. In addition, the expression of neutrophil activation markers (MMP9 and MPO) was comparable between the groups (Fig. 1A–F). Interestingly, PLWH had significantly decreased C2 but increased C5a levels compared to PLWoH, while there were no significant differences in the C3a and C9 levels, as shown in Fig. 1G–H. A recent study showed that complement activation is linked to the prevalence of comorbidities in PLWH.^21^ Based on altered C2 and C5a levels in PLWH, we further analyzed whether those levels could be attributed to differences in age and comorbidities among the PLWH. However, these two parameters did not impact C2 and C5a levels (Suppl. Fig. 1A–D). To further determine whether CMV co-infection may contribute to complement activation, we disaggregated PLWH and PLWoH samples based on CMV IgG serostatus and compared plasma C2 and C5a levels. Although the differences were not statistically significant, likely due to a small sample size, we observed a trend toward elevated C5a levels in CMV IgG-positive PLWH compared to CMV IgG-negative PLWH (Suppl. Fig. 1E–F).

### PLWH plasma displays an association of complement C2 and C5a with coagulation and inflammatory soluble markers

**Figure 2.**
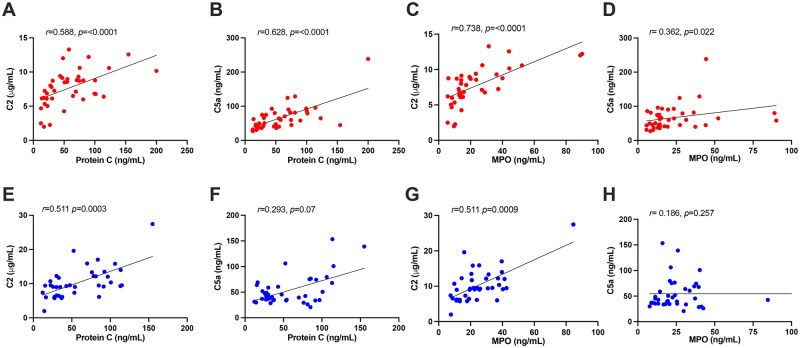
PLWH plasma displays a stronger positive association between complement C2 and C5a with protein C and MPO than PLWoH. Spearman correlation with protein C concentration with C2 and C5a in PLWH (A, B) and PLWoH (E, F) and MPO concentration with C2 and C5a in PLWH (C, D) and PLWoH (D, H).

C5a can induce neutrophils to form NETs^27^ and platelets to aggregate,^24^^,^^28^ providing evidence that elevated C5a levels facilitate sustained inflammation in various diseases, including arterial thrombosis and coronavirus disease 2019 (COVID-19).^29^ Given that the complement system is involved in regulating inflammation and coagulation, it is essential to determine whether altered levels of C2 and C5a are associated with neutrophil activation and coagulation. In both groups, C2 and C5a were positively correlated with Protein C, a natural anticoagulant (Fig. 2A, B, E, F). Additionally, MPO showed a positive correlation with C2 and C5a (Fig. 2C, D, G, H). Interestingly, these associations were more robust in PLWH than in PLWoH (Fig. 2). Spearman correlations were assessed between C2 and C5a and previous laboratory parameters, particularly inflammatory biomarkers associated with CVDs. Studies have shown that C2 deficiency is associated with increased susceptibility to infection and heightened inflammatory manifestations in patients with systemic lupus erythematosus (SLE),^30^ further prompting us to explore the relationship between C2 and inflammatory markers. Interestingly, C2 was significantly positively correlated with inflammatory markers, including serum amyloid A (SAA), serum amyloid P (SAP), interleukin-1 beta (IL-1β), and vascular endothelial growth factor (VEGF) in PLWH (Table S1).

A linear regression model analysis was conducted to determine whether demographics were predictive variables of C2 and C5a levels. Table S2 demonstrated that HIV status is a predictive variable of C2 and C5a levels, showing a significant negative beta-coefficient for HIV status with C2 levels but a significant positive beta-coefficient for HIV status with C5a levels. Overall, the data demonstrate that complement dysregulation in PLWH is positively correlated with increased levels of inflammation and coagulation markers in these individuals. HIV status may serve as a predictive factor for C2 and C5a levels.

### C5a showed increased NETosis on healthy neutrophils in PLWH’s plasma

**Figure 3.**
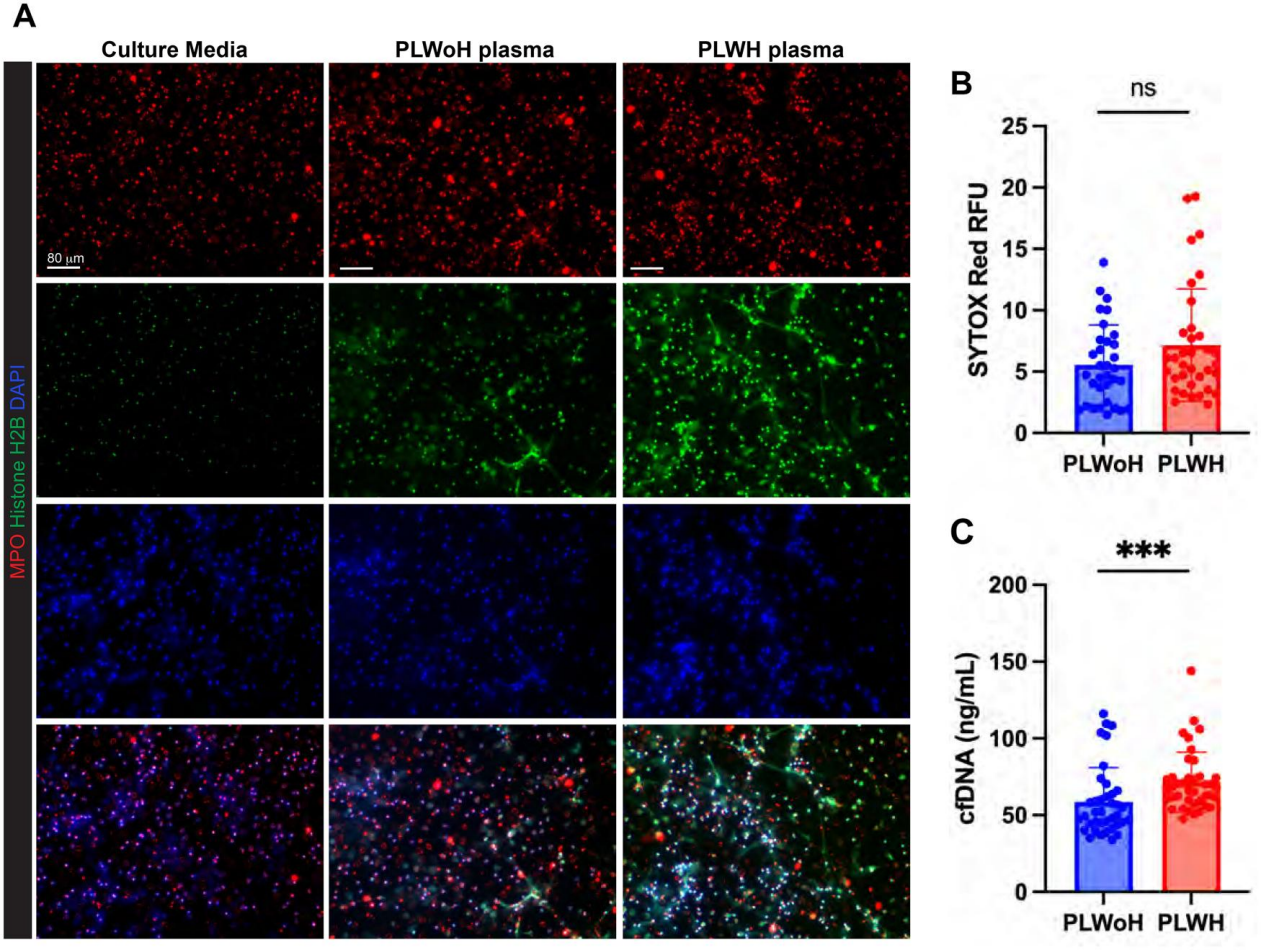
PLWH’s plasma treatment of healthy neutrophils promoted increased NET formation. (A) NET formation was visualized by immunofluorescence staining with Histone H2B (green, extracellular DNA), and MPO (red, extracellular DNA). NET formation was increased in healthy neutrophils treated with PLWH’s plasma, compared to PLWoH plasma. (B, C) Quantification of NETs using SYTOX Red Dead stain and the Quant‐iT PicoGreen dsDNA assay. ns; non-significance, ***P ≤ 0.001.

It has been demonstrated that C5a independently acts as a neutrophil chemoattractant and NET stimulus.^24^^,^^25^ Therefore, we hypothesized that PLWH plasma would promote NET formation due to increased levels of soluble proteins, including C5a. To assess whether plasma from PLWH has an increased tendency to trigger NETs, we utilized blood neutrophils isolated from healthy volunteers and cultured them in serum-free media supplemented with PLWH plasma or PLWoH plasma, respectively, for 3 h. NETs were visualized by immunofluorescence staining of anti-histone H2B and MPO antibodies. In the presence of plasma, regardless of HIV status, there appeared to be an increase in MPO expression (Fig. 3A). Interestingly, neutrophils treated with PLWH plasma resulted in even greater NET formation compared to PLWoH plasma (Fig. 3A).

**Figure 4.**
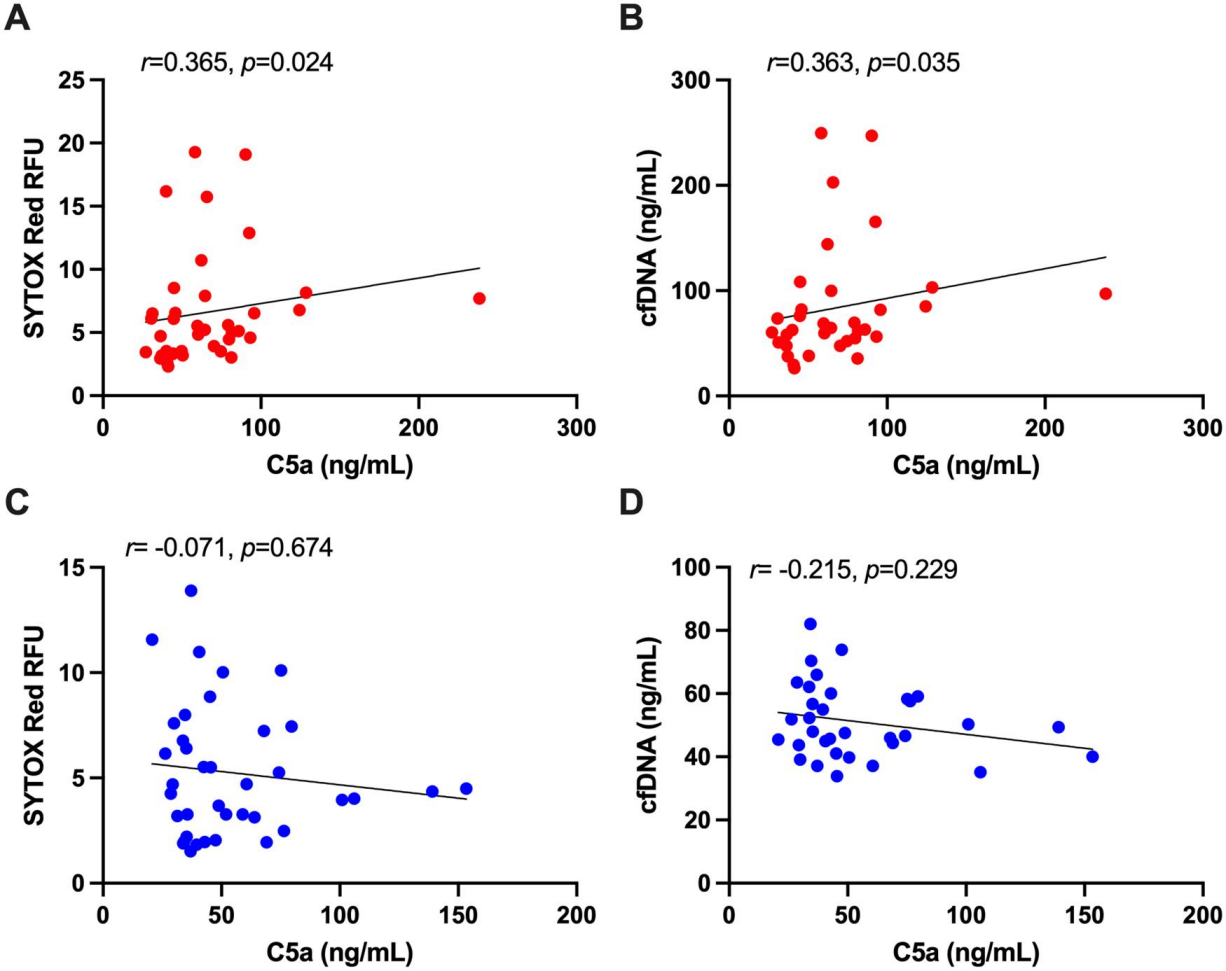
Complement C5a levels in PLWH plasma are associated with NETosis from healthy neutrophils. Spearman correlation analysis was performed between complement C5a levels and NETosis markers, including SYTOX red and cfDNA, in PLWH (A, B) and PLWoH (C, D), showing a significant positive correlation between parameters in PLWH but no association in PLWoH.

**Figure 5.**
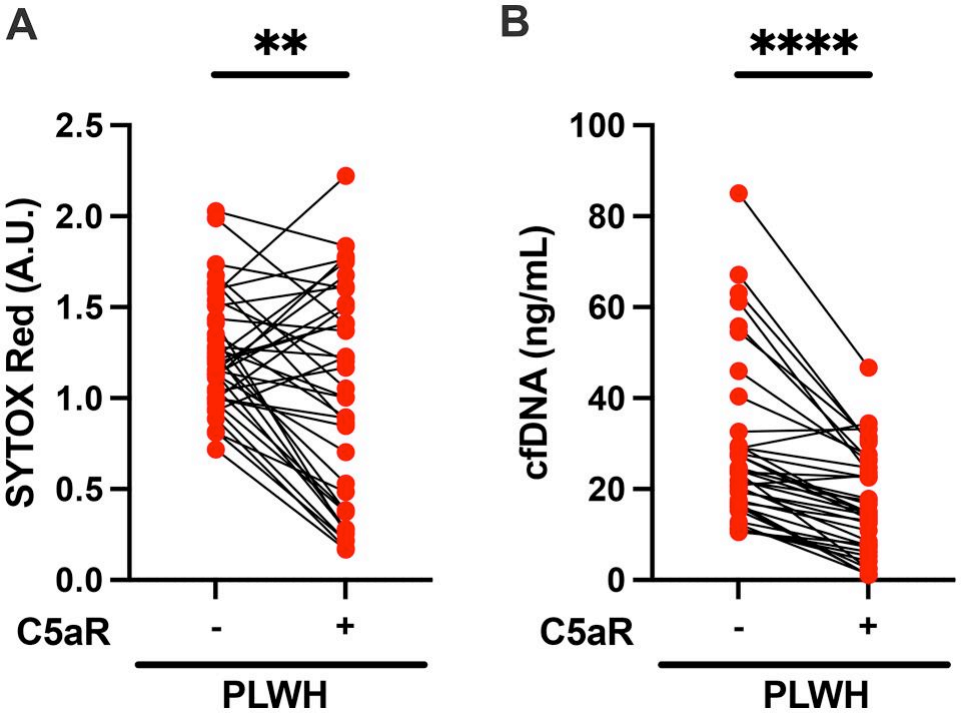
C5a/C5aR pathway blockade prevents NET formation induced by plasma from PLWH. (A, B) Healthy neutrophils were pre-treated with C5a antibodies (0.1 µg/ml) for 30 mins and then treated with PRP from PLWH (*n* = 38) for 3 h, NET formation was quantified using SYTOX Red Dead stain (A) and the Quant‐iT PicoGreen dsDNA assay (B). The Mann–Whitney test for paired data was used. ns; non-significance, **P ≤ 0.01, ****P ≤ 0.0001.

To further corroborate this observation and to quantify NETs in the culture conditions, we measured the fluorescence intensity of SYTOX Red dye in the cells, which serve as an indicator of NETosis, a programmed cell death, and cfDNA content in the culture supernatant using PicoGreen assay at the end of the neutrophil culture. The SYTOX Red fluorescence intensity trended higher with PLWH plasma treatment, however, this was not statistically significant (Fig. 3B). PLWH plasma-treated neutrophils produced a significantly increased amount of DNA compared to PLWoH plasma-treated neutrophils (Fig. 3C). Furthermore, there was a significant positive correlation of C5a with SYTOX dye (*r* = 0.365, *P* = 0.024) and cfDNA (*r* = 0.363, *P* = 0.035) in PLWH plasma (Fig. 4A, B). In contrast, no significant correlation was shown in PLWoH plasma (Fig. 4C, D). These results suggest that C5a levels in the plasma may act as a NET-stimulating factor in PLWH. Next, to determine if the C5a/C5aR axis regulates NET formation in chronic HIV, we performed ex vivo NET assays using a C5a receptor (C5aR) blocking antibody. The results showed that C5aR blockade led to a marked reduction in both SYTOX Red and cfDNA levels (Fig. 5A, B), providing direct evidence that elevated levels of C5a in HIV plasma activate neutrophil NET formation via the C5a/C5aR pathway.

### Altered platelet activation and coagulation marker associations in PLWH

**Figure 6.**
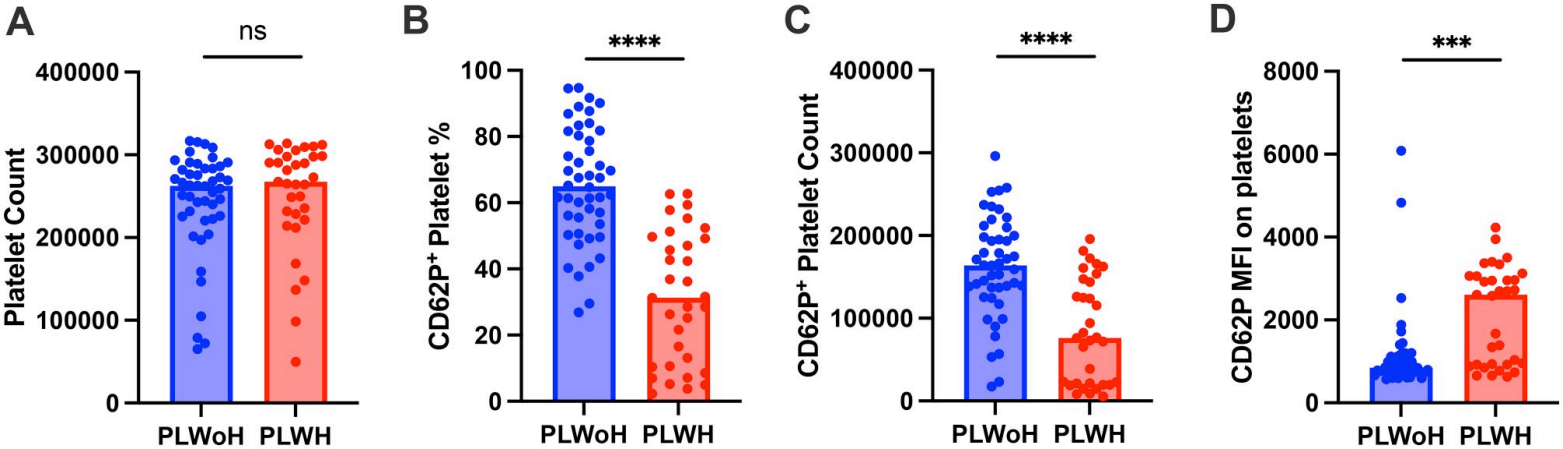
Comparison of platelet counts and activation in PLWoH and PLWH plasma samples by flow cytometry. (A–D) Flow cytometry analysis of (A) platelet count, (B) CD62P^+^ (platelet activation marker) platelet percent, (C) CD62P^+^ platelet count, (D) CD62 MFI. ns; non-significance, ***P ≤ 0.001, ****P ≤ 0.0001.

The plasma samples tested for ex vivo NET assay were platelet-rich, and platelets are known to modulate NET formation through their adhesion and activation.^31^^,^^32^ Therefore, we next performed flow cytometry analysis to assess platelet activation by measuring P-selectin (CD62P) expression on platelets (CD41^+^ cells) present in the plasma samples from the study cohort. Although platelet numbers were comparable between the groups (Fig. 6A), the percentages and numbers of activated platelets were significantly reduced in PLWH compared to PLWoH plasma (Fig. 6B, C). Interestingly, the mean fluorescence intensity (MFI) of CD62P expression was significantly elevated in PLWH, compared to PLWoH (Fig. 6D). These data suggest that PLWH display strong platelet activation although the activated platelet numbers are relatively low. To further assess whether coagulation regulation is altered in PLWH, we examined the associations among coagulation markers in both groups. Protein C and tissue factor (TF) represent opposing arms of the coagulation pathway,^33^ and we observed a significant negative correlation between protein C and vWF-A2 (*r*= −0.669, *P* = 0.001) and TF (*r*= −0.480, *P* = 0.028) in PLWoH (Table S3). ADAMTS13, a metalloprotease involved in cleaving vWF multimers, positively correlated with vWF-A2 and tissue factor,^34^ and protein C levels were negatively correlated with ADAMTS13 (*r*= −0.582, *P* < 0.001) in PLWoH, while we observed negative correlations between Protein C and other procoagulant markers (such as vWF-A2 and tissue factor) in PLWoH. However, PLWH did not show any significant correlations except for ADAMTS13 with TF (*r* = 0.734, *P* < 0.001) (Table S3). The absence of this association in PLWH suggests that the functional coordination within the coagulation cascade may be altered in PLWH.

### Treatment of healthy neutrophils with platelet-poor plasma from PLWH reduced lytic NET formation

**Figure 7.**
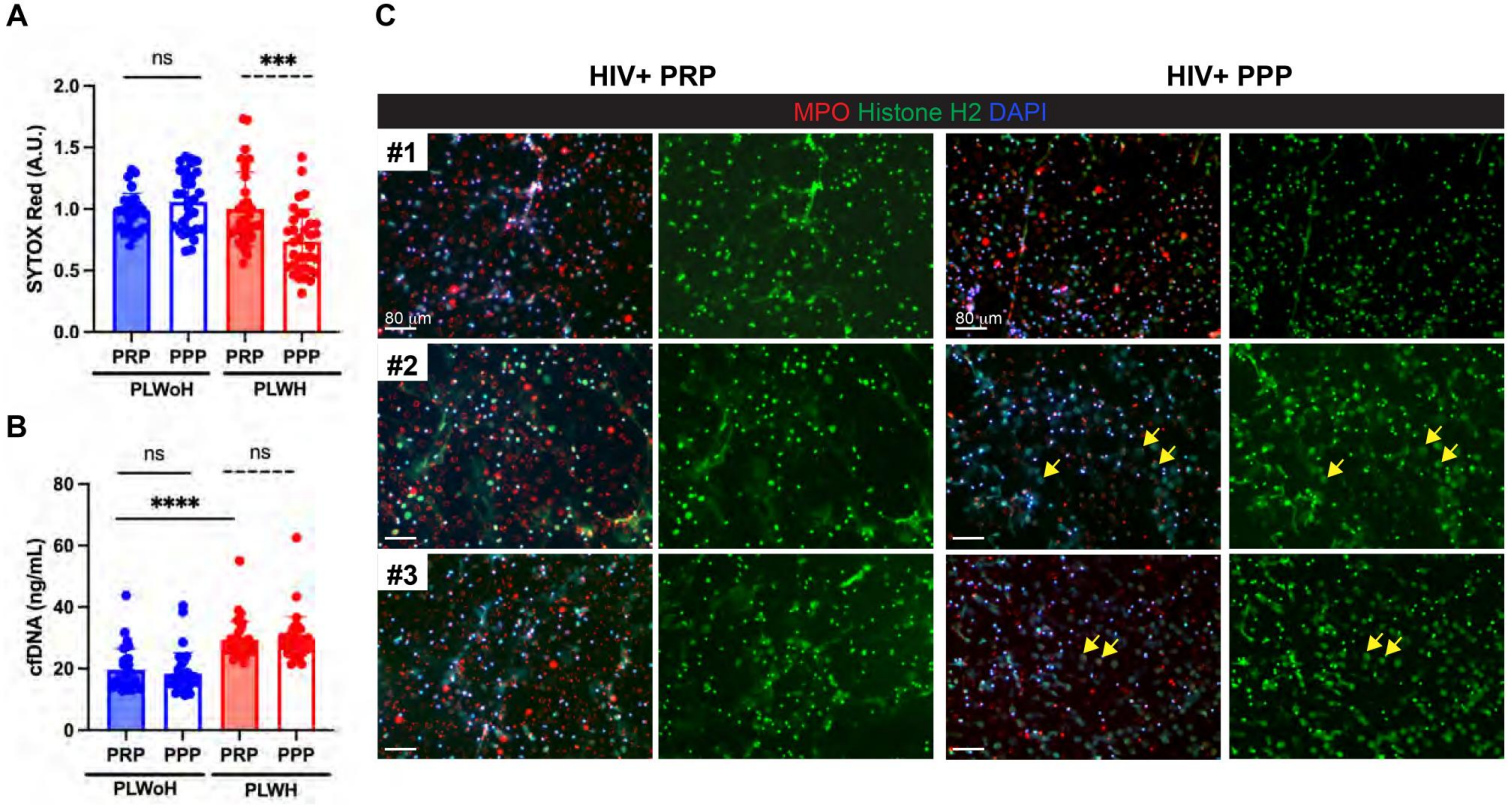
Effect of PRP and PPP from PLWH and PLWoH on *ex vivo* NET formation. (A, B) After 3 h incubation of healthy neutrophils treated with either PRP or PPP from PLWH (n = 34) and PLWoH (n = 32), NET formation was quantified using SYTOX Red Dead stain (A) and the Quant‐iT PicoGreen dsDNA assay (B). (C) Representative immunofluorescence image of NETs showed more intact cells positive for NET markers, indicated by the yellow arrow in the PPP treatment compared to the PRP treatment. ns; non-significance, ***P ≤ 0.001, ****P ≤ 0.0001.

A study showed that platelets from P-selectin^−/−^ mice failed to promote NETs.^32^ Also, NET formation was enhanced in mice engineered to overproduce soluble P-selectin,^32^ indicating that platelet P-selectin, in either cellular or soluble form, primes neutrophils for NET formation. Previously, we demonstrated that low-density granulocytes (LDGs) from PLWH exhibit increased NET formation.^23^ These results, derived from the same clinical cohort, provided the basis for investigating upstream drivers of NET dysregulation in the current study. Building upon that work, we sought to determine whether platelet activation contributes to the heightened NET formation observed in PLWH. To address this, we conducted ex vivo NET formation assays using platelet-rich plasma (PRP) and platelet-poor plasma (PPP) from PLWoH and PLWH. Overall SYTOX Red levels were not significantly different between PRP and PPP in PLWoH (Fig. 7A), suggesting that platelet depletion does not substantially impact NET formation. Similar to the SYTOX Red results, there was no significant difference in DNA concentration between PRP and PPP in PLWoH, and their levels were also comparable in PRP and PPP treatments from PLWH (Fig. 7B). Furthermore, NET release as indicated by cfDNA levels in culture medium was substantially lower in PLWoH plasma compared to PLWH plasma samples, consistent with findings shown in Fig. 3C. When neutrophils were treated with PPP from PLWH, the fluorescence intensity of SYTOX dye was significantly decreased compared to PRP treatment (Fig. 7B). Several studies have shown that non-lytic NETs, also referred to as vital NETs, can occur independently of cell death.^35^ This process occurs rapidly (5–60 min) compared to lytic NET formation, which takes place over 3–4 h.^36^ Our observations suggest PPP treatment induces non-lytic NETs, evidenced by reduced SYTOX dye staining, while cfDNA levels were comparable between PRP and PPP treatments. In an ex vivo NET assay with PRP and PPP treatment, we found that both conditions led to NET formation. Interestingly, the CitH3 staining pattern in PPP-treated neutrophils was diffuse and colocalized with MPO, as indicated by the yellow arrow. However, these staining patterns were not detected in PRP treatment (Fig. 6C). These results suggest that PPP treatment preferentially induces non-lytic NETs.

## Conclusion

Although the complement system plays a vital role in innate and adaptive immune responses, dysregulated complement activation has been linked to the development of inflammatory diseases. Altered plasma levels of complement components and factors in PLWH and their association with the risk of comorbidities have been suggested.^21^ However, how complement dysregulation contributes to chronic inflammation and thrombogenicity in PLWH remains largely unexplored. Our study found altered levels of C2 and C5a in virally suppressed PLWH and their associations with markers of inflammation and coagulation. HIV status was a significant predictor of C2 and C5a levels. Furthermore, increased P-selectin expression on platelets in the plasma samples from PLWH and treatment of healthy neutrophils with PLWH plasma induced NET formation, which was abrogated by C5a-C5aR blockade.

The decreased plasma C2 levels observed in our study are consistent with findings reported by Ivan Vujkovic-Cvijin et al. and this reduction may be partially attributed to the significant downregulation of C1r.^21^ Notably, the association between C2 and ADAMTS13 was weaker in the PLWH group than in the PLWoH group, suggesting that the coagulation cascade through C2-mediated complement pathways might not be tightly regulated in PLWH.

People with C2 deficiency are at increased risk of developing autoimmune disorders, including SLE,^37^^,^^38^ and are also highly susceptible to recurrent bacterial infections.^39^ In SLE patients, deficiencies in C1r and C1s are associated with increased disease susceptibility and enhanced proinflammatory responses, although such deficiencies are very rare.^40^ We observed significant positive correlations between C2 and several inflammatory and coagulation markers in PLWH. These findings highlight a complex interplay between complement components and inflammation in HIV. Further investigation is needed to elucidate the mechanisms underlying decreased C2 levels, including whether this is associated with reduced C1r/C1s expression or increased C2 consumption in chronic HIV infection. In addition, the role of C2 in chronic HIV requires further investigation.

C5a is a potent downstream chemoattractant and pro-inflammatory protein,^12^^,^^13^ suggesting that the mechanism of dysregulated inflammation may be caused by the activation of the alternative pathway instead of the classical and lectin pathway, as C2 levels, which are associated with these pathways, were decreased in PLWH. Studies have demonstrated that the excessive production of C5a can contribute to pathogenic proinflammatory and angiogenic responses in various diseases, including COVID-19 and sepsis.^19^^,^^41^ Indeed, C5a can induce neutrophils to form NETs and promote thrombosis through neutrophil and platelet activation.^24^^,^^42^ Interestingly, we observed stronger associations of C5a with Protein C and MPO in PLWH. In the ex vivo NET assay, increased C5a levels in PLWH plasma were positively correlated with DNA levels and fluorescence intensity of SYTOX-Red. Furthermore, C5a/C5aR blockade using a C5aR antibody significantly inhibited NET formation induced by PLWH plasma in neutrophils. These data suggest that higher C5a levels in circulation could contribute to persistent inflammation and further augment neutrophil activation and NET formation via C5a/C5aR pathway. Lastly, HIV status was a strong predictive variable for the concentration of C2 and C5a, further supporting the importance of elucidating the mechanisms of the complement cascade activation and its functional roles in NACM.

Studies have shown elevated expression of C3aR and C5aR on platelets, neutrophils, and other myeloid lineages in coronary artery disease compared to healthy controls.^43^^,^^44^ Considering that C5aR may play an essential role in the inflammatory response and that there is therapeutic potential in targeting C5aR in various diseases, including lung inflammation and COVID-19,^29^^,^^42^ it will be important to investigate whether PLWH display altered C5aR expression on immune cells. This may provide a better understanding of the regulatory mechanisms of inflammation and coagulation mediated by the C5a/C5aR axis in PLWH.

Other evidence indicate that platelets play opposing roles in chronic HIV infection and contribute to the development of comorbid conditions.^45^ Previous studies have shown that platelets in PLWH exhibit increased activation phenotypes,^45–47^ although the level of CD62P^+^ platelets can also be affected by protease inhibitors.^48^ Interestingly, stimulation of platelets from PLWH with thrombin ex vivo has been shown to increase the level of CD62P expression compared to healthy controls, which may be a result of higher levels of in vivo platelet activation.^49^ Although platelet count was comparable between PLWH and PLWoH, the intensity of CD62P expression was drastically increased in PLWH, suggesting that increased platelet activation in PLWH and the direct interaction of platelets with neutrophils are essential for NET formation. Interestingly, ex vivo NET assays verified that soluble factors in plasma from PLWH substantially impact non-lytic NET formation. This non-lytic NET formation has been implicated in various human diseases, including sepsis^31^^,^^50^ and SLE.^51^ Further work is needed to identify altered soluble proteins involved in the non-lytic NET formation in PLWH. Additionally, mechanistic studies are needed to explore the role of the complement cascade and its relationship with platelets and neutrophils. Such investigations may also help evaluate the therapeutic potential of targeting the complement system to modulate inflammation and immune activation in HIV.

The study has several limitations. including its small sample size. Furthermore, PLWH did not exhibit a significant difference in co-morbidities compared to PLWoH, suggesting that this cohort may not be fully representative of PLWH at increased risk for NACM. We analyzed selected complement components, which limits our ability to fully understand the dysregulation of the complement system in chronic HIV infection. Measuring C3a levels alone may provide a limited view of C3 activation. Future studies will incorporate additional C3 activation fragments, such as iC3b and C3dg, to more comprehensively assess complement activation and regulation. A more systematic and expanded analysis of the complement cascade, encompassing both activating components and their regulatory inhibitors, will be necessary to fully elucidate complement dynamics and dysfunction in the context of chronic HIV infection.

In summary, our study provides evidence of the association between C2 and C5a levels and inflammation and coagulation markers, as well as HIV status. Furthermore, treatment of neutrophils with plasma from PLWH demonstrated an increased capacity to promote NETs through the C5a/C5aR axis, potentially contributing to inflammation and coagulation in PLWH.

## Supplementary Material

vkaf227_Supplementary_Data

## Data Availability

The data underlying this article are available from the corresponding author upon reasonable request.
