# Supplementary material for: Dysregulation of complement components associated with inflammation and coagulation in virally suppressed people living with HIV
Source: J Immunol. 2025 Sep 15;214(11):2871–80. doi: 10.1093/jimmun/vkaf227 (PMC12453596; doi:10.1093/jimmun/vkaf227)
Supplement: vkaf227_Supplementary_Data [file vkaf227_supplementary_data.pdf]

SUPPLEMENTARY FIGURE

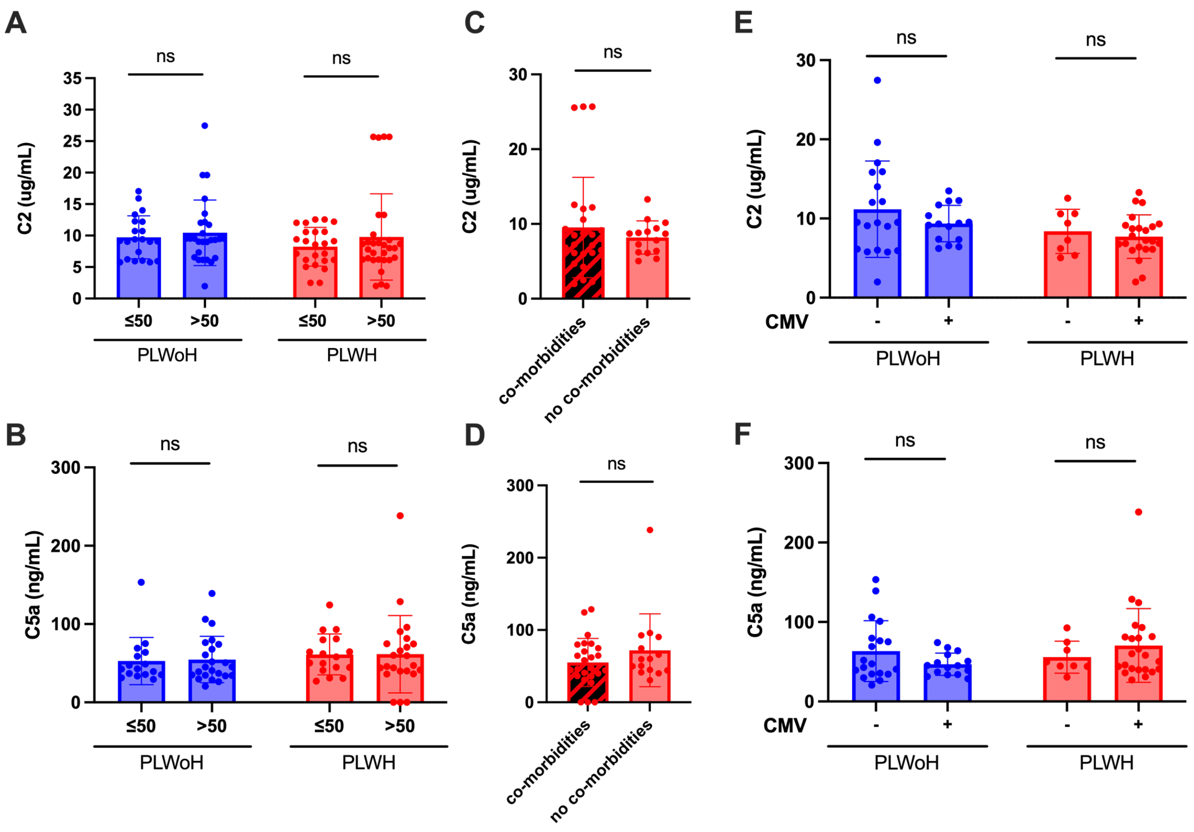

Supplementary Figure 1. Age, co-morbidities, and CMV serostatus do not significantly impact plasma complement C2 and C5 levels in both PLWoH and PLWH. (A-B) Plasma concentrations of C2 (A) and C5a (B) were compared between PLWoH (n=39) and PLWH (n=40), stratified by age (≤50 vs. >50 years). (C-D) Comparisons of C2 (C) and C5a (D) levels among PLWH with and without co-morbidities. (E-F) C2 (E) and C5a (F) levels were compared between PLWoH (n=39) and PLWH (n=34), stratified by CMV IgG serostatus (negative vs. positive). Statistical analysis was performed using the Mann-Whitney U test.

SUPPLEMENTARY TABLE 1

Parameters	Analytes	PLWH	
		Rho	P-value
C2	SAA	0.504*	0.001
	SAP	0.470*	0.003
	IL-1β	0.403*	0.012
	VEGF	0.374*	0.021

Supplementary Table 1. Complement C2 from PLWH plasma is associated with chronic inflammatory markers. Spearman correlation was done on complement C2 and C5a with chronic inflammatory and CVD markers, and a significant positive correlation was shown only in complement C2 levels with chronic inflammatory markers: SAA, SAP, IL-1β, and VEGF.

SUPPLEMENTARY TABLE 2

Independent Variable: Demographics	Dependent variable: logC2		Dependent variable: logC5a	
	Linear Regression			
	Beta-coefficient	P-value	Beta-coefficient	P-value
**HIV status**	-0.285*	0.012	0.261*	0.024
**Age**	0.037	0.740	0.069	0.547
**Male**	-0.134	0.235	-0.148	0.198
**BMI**	-0.006	0.960	0.038	0.749
**White**	-0.100	0.389	-0.099	0.403

Supplementary Table 2. HIV status is a predictive variable for complement C2 and C5a concentrations in PLWH plasma. A linear regression model was done on independent variables: HIV status, age, sex, BMI, and ethnicity, with the concentration of C2 and C5a levels showing a negative correlation with HIV status and C2 concentration levels, and a positive correlation with HIV status and C5a concentration levels.

SUPPLEMENTARY TABLE 3

Parameters	Analytes	PLWoH (n=39)		PLWH (n=40)	
		Rho	P-value	Rho	P-value
ADAMTS13	vWF-A2	0.768	<0.0001	0.205	0.349
	TF	0.764	<0.0001	0.734	<0.0001
	Protein C	-0.582	<0.0001	-0.262	0.102
Protein C	vWF-A2	-0.669	0.001	-0.283	0.191
	TF	-0.480	0.028	-0.140	0.391
	ADAMTS13	-0.582	<0.001	-0.283	0.102

Supplementary Table 3. Coagulation functionality difference between plasma in PLWoH and PLWH. Spearman correlation of coagulation regulation markers with coagulation activation markers in PLWoH (n=39) and PLWH (n=40). There is a more significant correlation between coagulation regulator markers and coagulation activation markers, inflammation markers, and complement protein markers in PLWoH than in PLWH.
